# The Role of Cancer-Associated Fibroblasts in Tumor Progression

**DOI:** 10.3390/cancers13061399

**Published:** 2021-03-19

**Authors:** Rushikesh S. Joshi, Samanvi S. Kanugula, Sweta Sudhir, Matheus P. Pereira, Saket Jain, Manish K. Aghi

**Affiliations:** 1 School of Medicine, University of California, San Diego, La Jolla, CA 92092, USA; rsjoshi@health.ucsd.edu; 2 Northwestern University, Chicago, IL 60201, USA; samanvikanugula2022@u.northwestern.edu; 3 Icahn School of Medicine at Mount Sinai, New York, NY 10029, USA; sweta.sudhir@icahn.mssm.edu; 4 School of Medicine, University of California, San Francisco, CA 94143, USA; matheus.pereira@ucsf.edu; 5 Department of Neurological Surgery, University of California, San Francisco, CA 94143, USA; saket.jain@ucsf.edu

**Keywords:** cancer, fibroblasts, mesenchymal, invasion, metastasis, tumor microenvironment

## Abstract

**Simple Summary:**

As our knowledge of cancer as a complex organ comprising tumor cells as well as surrounding cells within the microenvironment continues to grow, it is imperative to consider how the microenvironment may be supporting the cancer and promoting tumor progression. One aspect of the microenvironment that has gained significant interest over the past decade are cancer-associated fibroblasts, which have been implicated in diverse oncogenic roles including cancer invasion and metastasis, resistance to existing cancer therapeutics, angiogenesis, and tumor proliferation. The identification of cancer-associated fibroblasts and the pathways through which they promote tumor progression will allow us to target a specific subset of cells within the cancer niche in order to augment existing cancer therapies and possibly develop novel methods. In this review, we discuss the different markers that have been used to identify cancer-associated fibroblasts in various cancer contexts as potential therapeutic targets and discuss the role that cancer-associated fibroblasts play in enhancing cancer malignancy.

**Abstract:**

In the era of genomic medicine, cancer treatment has become more personalized as novel therapeutic targets and pathways are identified. Research over the past decade has shown the increasing importance of how the tumor microenvironment (TME) and the extracellular matrix (ECM), which is a major structural component of the TME, regulate oncogenic functions including tumor progression, metastasis, angiogenesis, therapy resistance, and immune cell modulation, amongst others. Within the TME, cancer-associated fibroblasts (CAFs) have been identified in several systemic cancers as critical regulators of the malignant cancer phenotype. This review of the literature comprehensively profiles the roles of CAFs implicated in gastrointestinal, endocrine, head and neck, skin, genitourinary, lung, and breast cancers. The ubiquitous presence of CAFs highlights their significance as modulators of cancer progression and has led to the subsequent characterization of potential therapeutic targets, which may help advance the cancer treatment paradigm to determine the next generation of cancer therapy. The aim of this review is to provide a detailed overview of the key roles that CAFs play in the scope of systemic disease, the mechanisms by which they enhance protumoral effects, and the primary CAF-related markers that may offer potential targets for novel therapeutics.

## 1. Introduction

Several recent studies have uncovered that cancer is not just a mass of malignantly transformed cells but is instead an organ harboring complex interplay between tumor cells and cells in their microenvironment. As efforts have moved in this direction, the tumor microenvironment (TME) with its diverse range of cell types, and the extracellular matrix (ECM), which provides a structural component to the TME, are of renewed interest [1,2,3,4,5,6]. Components of the TME, such as cancer-associated fibroblasts (CAFs), which are activated fibroblasts in the tumor stroma, have been implicated in cancer malignancy and tumor progression [7]. CAFs have been found to contribute to non-restricted growth, angiogenesis, invasion, metastasis, and therapy resistance [8]. Notably, CAFs have been identified in numerous systemic cancers in which they embody unique roles, illustrating their heterogeneity [9]. Cancers where CAFs have definitively been characterized span solid tumors and carcinomas across almost every physiologic system, including head and neck [8,10,11], breast [12,13,14], lung [15,16,17,18], skin [19,20,21], gastrointestinal (GI) and biliary tract [22,23,24], and genitourinary (GU) cancers [25,26]. To date, there has not been any indication that CAFs are implicated in lymphoid and hematopoietic cancers, and there is sparse, but growing evidence alluding to the presence of CAFs in central nervous system (CNS) cancers such as glioblastoma multiforme (GBM) [27,28,29].

Although CAFs were previously considered to be a homogenous population, recent evidence indicates that understanding the heterogenous functionality of CAFs and CAF-related markers in different systemic cancers can lead to cutting-edge advances in personalized, targeted cancer therapy [30]. In this review, we provide a detailed overview of the key roles that CAFs play in the scope of systemic disease, the mechanisms by which they enhance protumoral effects, and the primary CAF-related markers that may offer potential targets for novel therapeutics. 

## 2. Cancer-Associated Fibroblast (CAF) Markers

CAFs have been identified in most studies through their expression of “CAF markers”, such as fibroblast activation protein alpha (FAP) and alpha smooth muscle actin (α-SMA), markers that are felt to separate CAFs from normal fibroblasts [31]. What distinguishes CAFs from normal fibroblasts is that they interact with tumorigenic cells in the TME and persist in a hyper-activated state that enhances cancer progression through several different pathways. Unfortunately, the expression of CAF markers is extremely heterogeneous and varies strongly between different CAF subpopulations as described below (Table 1). For example, some common CAF markers that have been identified include alpha smooth muscle actin (α-SMA), vimentin, fibroblast activation protein (FAP), fibroblast-specific protein 1 (FSP1), and platelet-derived growth factor receptor alpha/beta (PDGFR-α/β) [31,32,33,34].

However, the problem that persists is that several of these markers have their own drawbacks, including low specificity and limited experimental studies in the current literature. In addition, it is unclear how the anatomic location and cancer context affects the expression of CAF markers, as CAFs in the stroma of a particular cancer may express a completely different genetic profile and set of markers than CAFs in a different cancer/anatomical context. To navigate the lack of a ubiquitous marker, ongoing studies have focused on identifying novel methods of selecting CAFs on the basis of cellular function, as well as attempts to identify novel CAF markers. Due to their higher contractility, contraction assays have previously been used to differentiate between CAFs and normal fibroblasts [65]. Three-dimensional hydrogel assays, using substances such as collagen or matrigel, are another way of characterizing CAFs and their subtypes in a functional manner [66,67,68]. It is imperative to continue developing novel methodologies for isolating CAFs and studying their function, in order to allow collaboration across different cancer types. The heterogeneity inherent to CAF presentation, may in turn allow for more specific and targeted therapies to be developed in a more individualized manner for patients with specific cancer types.

## 3. The Origin of CAFs

**Figure 1:**
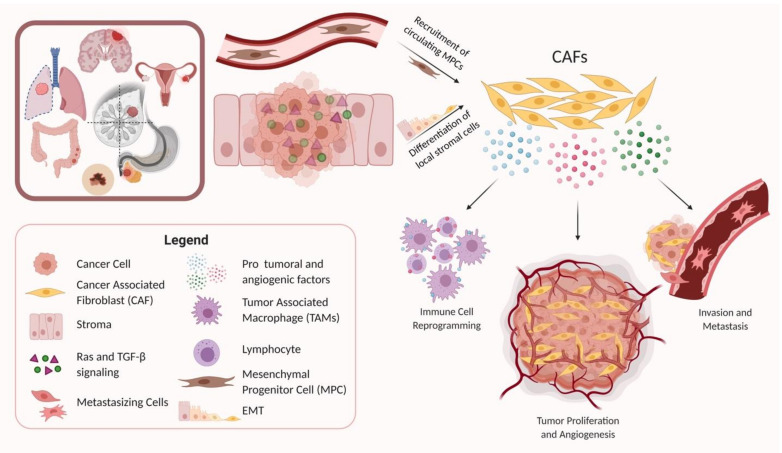
This diagram represents how CAFs are recruited into the tumor microenvironment (TME) or activated through various methods. Once activated, CAFs exert several protumoral effects including immune modulation of the TME, tumor cell proliferation and angiogenesis, and promotion of invasion and metastases, among others. TGF-β—transforming growth factor beta, EMT—epithelial mesenchymal transition

Several different cellular origins of CAFs have been demonstrated in studies using preclinical cancer models. Some of these studies have suggested that CAFs differentiate from local cells in the tumor microenvironment, while other studies have suggested that CAFs arise from the recruitment of circulating progenitors that differentiate into CAFs upon arrival in the TME (Figure 1) [69,70,71,72]. Local cells in the TME that have been demonstrated to differentiate into CAFs in mouse models of cancer included local fibroblasts, endothelial cells, and vascular mural cells [73,74,75]. The process of differentiation of local cells into CAFs often includes epithelial–mesenchymal transition (EMT), as seen by the transdifferentiation of myofibroblasts from epithelial cells, or the acquisition of mesenchymal morphology with loss of E-cadherin by activating Ras and transforming growth factor beta (TGF-β) signaling [76,77]. Other routes of CAF activation include differentiation from resident fibroblasts and mesenchymal stem cells by a host of myriad cancer cell-produced factors, and endothelial to mesenchymal transition (EndMT) [70,78,79]. In contrast, some studies have implicated circulating bone marrow-derived cells, most likely mesenchymal stem cells, as being recruited into tumors where they can differentiate into CAFs [12,17,80,81].

## 4. CAF Promotion of Tumor Growth and Maintenance of Stemness

One of the primary mechanisms by which CAFs contribute to tumor malignancy is by promoting tumor growth. Tumor cell proliferation is an essential step for tumorigenesis and almost always entails the recruitment of supportive stromal cells to work in a synergistic manner. It is now widely accepted that cancer cells and stromal cells such as CAFs dynamically co-evolve over the course of tumor progression. CAFs exist as part of the surrounding stroma in the TME and facilitate tumor growth through the secretion of growth factors, cytokines, and chemokines in addition to remodeling of the ECM. Tumor cells have been shown to interact reciprocally with CAFs by activating them, and then subsequently benefitting from their protumoral effects through secreted growth factors and cytokines. The tumor-supporting effect has been shown to result from CAF secretion of various factors including hepatocyte growth factor (HGF), transforming growth factor beta (TGF-β), stromal-derived factor 1 (SDF-1), interleukin 1β (IL-1β), PDGF, phosphatase and tensin homolog (Pten), and Sonic hedgehog (Shh), among others, and activation of their respective receptors [71,82]. In addition to promoting tumor cell proliferation, CAFs have also been shown to increase the stem cell-like properties of cancer cells, particularly through secretion of growth factors such as HGF. The maintenance of stem cell properties has recently been shown to be a significant contributing factor to tumor formation, and the stromal niche is believed to regulate the differentiation and proliferation of cancer stem cells by providing a supportive TME [69].

### 4.1. Lung Cancer

Due to their heterogeneity, CAFs have been shown to promote lung cancer growth through multiple, unique mechanisms across the lung cancer subsets including non-small cell lung carcinomas (NSCLCs), which consist of lung squamous cell carcinoma (LSCC) and adenocarcinoma. CAF markers that have been shown to propagate lung tumor growth include α-SMA, with osteopontin (OPN) being a marker of CAF activation in lung fibroblasts and also a key mediator of tumor progression [18,83]. Using patient-derived samples from normal human proximal bronchi and distal lung parenchyma, Pechkovsky et al. studied how CAFs in the lung parenchyma spontaneously express α-SMA, implicating α-SMA’s role in lung cancer development [83]. Meanwhile, OPN was found to have a higher expression in senescent fibroblasts and is considered one of the key mediators of senescent stroma promoting tumorigenesis [18,83]. Additionally, Zhou et al. found that CAF-conditioned media increased the proliferation, migration, and invasion of lung cancer cells, partially through increased expression of vascular cell adhesion molecule-1 (VCAM-1) compared to conditioned media from normal lung fibroblasts (NLFs) [84]. Their study demonstrated that VCAM-1 secreted from CAFs activated AKT and MAPK signaling pathways, enhancing tumor growth and invasion in a reversible manner (with VCAM-1 blocking antibodies). Relating to the maintenance of cancer stemness, Chen et al. demonstrated that CAFs that were isolated from lung cancer patients increased the expression of stemness factors such as Nanog and Oct4 when co-cultured with lung cancer cells through an IGF-II-mediated interaction [85]. This increase in Nanog and Oct4 expression was shown to be reversible by blocking IGF-II/IGF1R signaling, and similarly the increase in stem cell markers was not observed when lung cancer cells were co-cultured with normal fibroblasts.

### 4.2. Breast Cancer

CAF markers that have been associated with promotion of breast cancer tumor growth through several experiments include α-SMA, FAP, PDGFR-α, PDGFR-β, CD29, neural/glial antigen 2 (NG2), FSP1, vimentin, and podoplanin (PDPN) [36,37,86]. Jung et al. used microarray analysis to find that higher expression of FAP, PDPN, NG2, and PDGFR-β was discovered in adipose stroma, while higher levels of FSP1 were found in fibrous stroma [86,87]. In another study, Yu et al., by secreting TGF-β1, found that cancer-associated fibroblasts with α-SMA expression induced more aggressive phenotypes of breast cancer cells through EMT in a reversible manner, with enhanced cell–ECM adhesion, and migration/invasion [88].

### 4.3. Gastrointestinal (GI) Cancer

CAFs have been widely studied in numerous cancers of the GI system, from esophageal cancers, biliary tract cancers, all the way to colorectal cancers. Their function varies dramatically across the cancers, with a diverse range of effector functions. In a study by Sha et al., CAFs were identified on the basis of positive staining for FAP, α-SMA, FSP-1, and PDGFR-β, and intrahepatic cholangiocarcinoma (ICC) cells were observed to proliferate faster in CAF-conditioned media when compared to normal fibroblast conditioned media [57]. In addition, CAFs that were co-injected with tumor cells helped promote tumor growth dramatically in patient-derived xenograft (PDX) in vivo models when compared to ICC cells alone or ICC cells with normal fibroblasts. This study further corroborates results from Sirica et al., showing that α-SMA-positive CAF cells also promoted ICC tumor progression through paracrine signaling pathways including HGF, SDF-1, tenascin C (TN-C), and periostin (POSTN) [89]. With respect to hepatocellular carcinoma (HCC), Eunice et al. demonstrated that CAFs regulate liver tumor-initiating cells and found that the presence of α-SMA-positive CAF cells correlated with poor clinical outcome, corroborating earlier studies as well [56,90]. They were able to show that CAF-derived HGF regulates liver tumor-initiating cells via activation of FRA1 in an Erk1/2-dependent manner, highlighting the possibility of targeting the CAF-derived, HGF-mediated *c-Met*/*FRA1/HEY1* cascade as a therapeutic strategy for treatment of HCC.

In the context of colorectal cancers, Bai et al. showed that in colon cancers specifically, CAFs significantly promoted tumorigenesis and proliferation using both in vivo and in vitro models [24]. CAFs were identified on the basis of α-SMA, vimentin, and FAP expression, and were observed to secrete factors including fibroblast growth factor (FGF)-1 and FGF3 to promote tumorigenesis via the mitogen-activated protein kinases/extracellular signal-regulated kinases (MAPK/ERK) signaling pathway in vivo, and increased cell proliferation in vitro. Importantly, this effect was reversible with the addition of anti-FGF-1 or anti-FGF3 treatments. Additional CAF effects in colorectal cancers include maintenance of cancer cell stemness, as described by Liu et al. when CAF-conditioned media was observed to promote clonogenicity of colorectal cancer cells, which in turn conferred radioresistance through CAF-derived exosomes [91,92]. When exploring esophageal cancers, Zhao et al. demonstrated that CAFs expressing α-SMA enhanced progression of esophageal squamous cell carcinomas by promoting Shh expression, and notably this effect was partially reversible in vitro and in vivo by using cyclopamine to inhibit the Hedgehog signaling pathway [50].

### 4.4. Skin Cancer

In a novel study looking at non-melanoma skin cancer (NMSC), Cangkrama et al. identified cancer cell secretion of activin A, rather than TGF-β as a major activation factor for CAF cell differentiation into a protumoral phenotype through activation of a Smad2–mDia2–p53 signaling axis [19,93]. Their study demonstrated in PDX in vivo models and 3D organotypic models that cancer cells with high expression of activin A formed larger tumors and also had significantly higher invasion of the basement membrane layers, in addition to significantly increased stromal fibroblast proliferation rates. Additional contributors identified included increased secretion of active-matrix metalloproteinases (MMPs) such as MMP2 and MMP9. Conversely, Guo et al. identified α-SMA-positive CAF cells in melanoma cancer tissue that were activated by TRAF6, and promoted melanoma cancer growth, migration, and invasion as measured using CAF-conditioned media vs. normal fibroblast-conditioned media in vitro assays in addition to xenograft in vivo models [3].

### 4.5. Ovarian Cancer

CAFs similarly play a significant role in tumor progression in ovarian cancer. CAF markers that have been identified in ovarian cancer include α-SMA, FAP, FSP1, and FGF-1 [47,48]. Studies by Sun et al. showed that CAFs isolated from patient ovarian tissues promoted proliferation, migration, and invasion of ovarian cancer cells in culture studies. They further used immunocytochemistry analysis to discover that these protumoral effects are mediated through secretion of FGF-1 inducing activation of the MAPK signaling pathway and increased MMP3 expression [47]. 

### 4.6. Endometrial Cancer

CAF markers that have been identified in endometrial cancer include α-SMA, FSP1, FAP, and vimentin. CAFs isolated from endometrial cancer tissues were highly positive for α-SMA, FSP1, and FAP expression and moderately positive for vimentin expression, and were found to play diverse roles in endometrial tumor progression. Teng et al. sought to detect the functional effects of CAFs in the context of endometrial cancer progression, and using proliferation assays, the authors found that CAFs promote tumorigenesis in endometrial cancer via the SDF-1 α/CXCR4 axis in a paracrine - or autocrine-dependent manner. They further observed using PDX models that HEC-1B cells co-mixed with CAFs generated tumors of greater volume than the normal fibroblasts or HEC-1B cells alone [26].

### 4.7. Prostate Cancer

A study by Ortiz-Otero et al. used cell proliferation assays to demonstrate that when co-cultured with prostate cancer cells, reactive CAFs, defined as recently activated CAFs, which showed higher expression of α-SMA and FAP compared to differentiated CAFs, better supported tumor proliferation [32].

### 4.8. Renal Cell Carcinoma

Several studies have highlighted the protumoral effects exhibited by CAFs in renal cell carcinoma (RCC). CAF markers that have been identified in RCC include α-SMA, FAP, and POSTN. In particular, clear cell renal cell carcinoma (CCRCC) is a smaller subset of RCC, and studies by Errarte et al. and Zagzag et al. showed that CAFs may play a significant role in early phases of CCRCC development. The loss of the Von Hippel-Lindau (VHL) gene is a driver mutation event in CCRCC, which has been shown to induce overexpression of hypoxia-inducible factor 1 (HIF-1α), the accumulation of which increases expression of factors such as vascular endothelial growth factor (VEGF), SDF-1, and PDGF that recruit and active normal fibroblasts into CAFs from the TME. CAFs were demonstrated in co-culture experiments to promote RCC growth and proliferation and that the expression of SDF-1 and its receptor CXCR4 affected tumor cell proliferation and chemoresistance through interactions in the TME, offering a potential pathway for therapeutic intervention [45,46].

### 4.9. Bladder Cancer

CAF markers have been identified in urothelial bladder cancer and include α-SMA, FAP, CD90, vimentin, PDGFR-α/β, and MFAP5 [43,44]. Mezheyeuski et al. studied various CAF markers’ associations with survival and histopathological characteristics in patients with urothelial bladder cancer. They demonstrated that α-SMA, FAP, CD90, and PDGFR-α/β were involved in tumor progression in urothelial bladder cancer. A study investigating CAF signaling pathways in urinary bladder cancer found that CAFs exhibited greater expression levels of α-SMA, FAP, FSP1, and CD90 as opposed to normal fibroblasts. Vimentin was highly expressed in both normal fibroblasts and CAFs, and was deemed to not be a CAF-specific marker in urinary bladder cancer [44].

## 5. Pro-Angiogenic Effects of CAFs

Another modality through which CAFs promote tumor growth and malignancy is by driving angiogenesis through recruitment of endothelial cells and by increasing vascularization of tumors to enhance delivery of nutrients and growth factors. Studies have demonstrated CAF-promoted growth in xenograft models due to connective tissue growth factor (CTGF) expression, resulting in increased microvessel density, as well as recruitment of endothelial cells through expression of SDF-1/CXCL12 [94]. Aside from these direct effects on angiogenesis, CAFs have also been shown in numerous studies to indirectly affect tumor growth from the ECM through expression of MMPs such as MMP9 and MMP13 [21,95]. Both MMP9 and MMP13 have been shown to release active growth factors such as VEGF from the ECM to increase tumor angiogenesis. Despite their importance, MMPs are not restricted to CAFs, and as such other stromal cells also play integral roles as sources of angiogenesis-promoting factors.

### 5.1. Breast Cancer

CAFs were also found to promote angiogenesis through the recruitment of endothelial progenitor cells (EPCs) into breast carcinomas [96]. This function of CAFs was modulated by the CAF-secreted SDF-1, which is colocalized with α-SMA expression, accompanied with the loss of mDia2 protein expression, ultimately propagating tumor cell growth [88,96,97]. Additionally, CAFs were shown to secrete VEGF to assist in the formation of new blood vessels to facilitate tumor growth and expansion in breast cancer tissue [98].

### 5.2. Head and Neck Cancer

The role of CAFs has been well characterized in head and neck cancers, and Wang et al. demonstrated a relationship between CAFs and neoangiogenesis in nasopharyngeal carcinoma (NPC) [62]. In their study, CAFs were identified using immunohistochemistry for the α-SMA marker, as was stromal expression of SDF-1 and its receptor CXCR4. Significantly higher expression of α-SMA was observed in fibroblasts found in NPC stroma, as was immunoreactive intensities of SDF-1 and CXCR4 secreted by CAFs in NPC cells. Microvessel density was found to be higher in the stroma of NPC tissues as was the presence of CXCR4-positive and CD133/VEGFR-2-double-positive cells, indicating the presence of endothelial progenitor cells in both NPC cancer and stromal cells to enhance neoangiogenesis in a VEGF- and SDF-1 dependent manner.

### 5.3. GI Cancer

With respect to HCC, Liu et al. showed that CAFs in the peritumoral tissue and stroma exhibited high expression of α-SMA and CD90 (THY1), which correlated highly with HCC tissue expression of placental growth factor (PGF) [99]. An analysis of The Cancer Genome Atlas (TCGA) showed that high levels of PGF and CD90 are correlated with tumor angiogenesis markers (CD31, CD34, and CD105) and also predicted poor prognosis in HCC patients.

### 5.4. Skin Cancer

One of the distinguishing aspects of CAFs is the variability in surface markers used to identify them, as there is no ubiquitously expressed marker. As such, studies often utilize different markers in their experiments to identify and sort CAF cells. Erez et al. in their study opted to use PDGFR-α-positive fibroblast cells identified from squamous cell carcinoma tissue to investigate the proinflammatory role of CAFs [20]. Their study demonstrated that CAFs can contribute both directly through expression of genes such as CYR61 and OPN, but also indirectly through expression of inflammatory genes such as CXCL1, CXCL2, and CXCL5, which are chemoattractants for macrophages and neutrophils. Tumors injected with CAFs were found to be significantly more vascularized as evidenced by ultrasound imaging with contrast-enhanced micro-bubbles, exhibiting significantly higher blood vessel density and decreased tumor necrosis. Several of the inflammatory genes upregulated by CAFs in this study are known targets of NF-κB, and importantly this proinflammatory and angiogenic signature was reversible with knockdown of NF-κB expression.

### 5.5. Renal Cell Carcinoma

According to Errarte et al. and Zagzag et al., CAFs expressing α-SMA promoted angiogenesis in the RCC TME through secretion of SDF-1 in a novel angiogenic pathway. SDF-1 secretion was induced by hypoxic conditions where accumulation of HIF-1α promoted activation of normal fibroblasts into CAFs [45,46].

## 6. CAF Stimulation of Invasion and Metastasis

In addition to supplementing local tumor growth and progression, CAFs have also been shown to be integral components of the stroma for promoting increased cancer invasiveness at the site of the primary tumor and facilitating metastases to distant organs. Only a small portion of cancers end up forming clinically detectable metastases remote from the primary tumor, however, those that do generally comprise a significant component of cancer-related morbidity and mortality [82]. CAFs are able to stimulate cancer invasiveness through a combination of direct cell-to-cell interactions and secreted factors such as cytokines and chemokines, and inflammatory mediators, similar to how they enact their other protumoral effects [69]. Inherent to cancer invasion, is the necessity to disrupt the basement membrane in order to allow for cancer cell intravasation. Often, crosstalk between CAFs and cancer cells can result in modification of the ECM as well through perturbations of intrinsic scaffolding and cytoskeleton genes [100]. In addition to stiffening of cancer tissue and disruption of the basement membrane, stromal cells including CAFs have also been shown through co-cultured experiments to create invasive paths and make tracks in the stroma for cancer cells to migrate to other sites [101]. Invasive tracks are made through a combination of both proteolytic and structural modifications to the ECM by secretion of proteins such as collagens in addition to MMP enzymes. Beyond tumor invasion, CAFs can also play an integral role in the colonization of distant organs in metastatic disease, by creating a niche for tumor cells. CAFs have been shown to provide stromal support for disseminated cancer cells through a combination of environmental factors, including POSTN and TN-C proteins [102,103]. As described before, CAFs can induce POSTN production via TGF-β signaling to facilitate metastatic colonization of cancerous cells. TGF-β further promotes the metastatic paradigm by inducing EMT through paracrine signaling, allowing premalignant cells to acquire mesenchymal properties for invasion and metastasis. As a collective, MMPs, POSTN, TN-C, and other CAF-secreted molecules offer novel therapeutic targets for mitigation of cancer invasion and metastasis.

### 6.1. GI Cancer

A study by Sha et al. investigating the role of CAFs in ICC tumors showed that FAP-, PDGFR-β-, α-SMA-, and FSP-1-positive CAFs accelerated ICC tumor cell invasion and migration when cultured in CAF-conditioned media [57]. In the context of oral cancers, CAFs have also been implicated as protumoral effector cells in oral squamous cell carcinomas (OSCC). Importantly, Wang et al. demonstrated that silencing FAP inhibited growth and metastasis of OSCC cells both in vitro and in vivo, by inactivating the PTEN/PI3K/AKT and Ras-ERK signal pathways, which are known to regulate proliferation, migration, and invasion [61].

The role of CAFs has also been widely explored in human pancreatic cancers, where they have been found to contribute to cancer invasion and metastasis. In a study conducted by Wei et al., CAFs were identified from pancreatic cancer tissue on the basis of positive immunohistochemistry (IHC) for α-SMA, as well as immunofluorescence, showing high expression of α-SMA and FAP when compared to normal fibroblasts [53]. In their study, Wei et al. observed that SDF-1 secreted by CAFs stimulated malignant progression, migration, and invasion of pancreatic cells in vitro through paracrine induction of SATB-1 in pancreatic cancer cells. The interaction between CAFs and pancreatic cancer cells was found to be a positive feedback loop, with SDF-1 promoting SATB-1 expression in the cancer cells, which in turn contributed to the maintenance of CAF phenotypes in the stroma. In addition, overexpression of SATB-1 in cancer cells was found to contribute to the process of gemcitabine resistance. Other studies including one conducted by Awaji et al. demonstrated that secreted mediators such as PDGF and TGF-β also play significant roles in activating CAFs in the context of pancreatic ductal adenocarcinoma (PDAC), and sustained levels of TGF-β have been implicated in stiffening of the tumor tissue and TME, correlating highly with metastasis and poor survival [9]. With respect to gastric cancers, studies have also demonstrated a role of CAFs in promoting tumor progression, including an association with *Heliobacter pylori (H. pylori)* infection, which is one of the strongest risk factors for developing gastric carcinoma. Shen et al. demonstrated in their study that *H. pylori* infection increased VCAM1 expression in CAFs via activation of the JAK/STAT1 signaling pathway, and increased VCAM1 levels in turn were positively associated with tumor progression and poorer prognosis [104].

### 6.2. Lung Cancer

In the context of adenocarcinoma, studies have illustrated the involvement of multiple CAF-related markers. Studies by Neri et al. through collagen invasion assay models showed that knockdown of PDPN (CAF product) decreased invasion of CAFs and lung adenocarcinoma cells in the collagen matrix [40,105]. In another study, Li et al. used IHC to demonstrate that the enzyme FUT8 is overexpressed in CAFs and stimulates proliferation and invasiveness of NSCLC cells in the TME by modulating epidermal growth factor receptor (EGFR) expression [15]. Through IHC, they also found that mRNA levels of the markers α-SMA and FAP in CAFs were significantly higher than those in NLFs.

### 6.3. Endocrine/Neuroendocrine Cancers

Thyroid carcinomas comprise several different histopathological cancers that present with different levels of aggressiveness. While studies have previously acknowledged a role for CAFs in the papillary thyroid carcinoma variant, Minna et al. investigated the presence of CAFs in papillary thyroid carcinoma, poorly differentiated thyroid carcinoma, and anaplastic thyroid carcinoma [64]. In their study, CAFs were detected using immunostaining for α-SMA across all thyroid tumor types and found to localize preferentially in the stroma along the tumor invasive front. Expression of α-SMA-positive CAFs was found to be closely associated with stromal expression of COL1A1, whereas lysyl oxidase (LOX) was expressed by adjacent thyroid tumor cells. These findings suggest that CAFs may be implicated in the local invasion of thyroid carcinomas. In a similar context, Hashimoto et al. found that CAFs, once again defined by their positive staining for α-SMA, were dominant in stage 4 neuroblastoma tissue, and density of CAFs as defined by area correlated significantly with aggressive phenotypes as defined by clinical stage, MYCN amplification, bone marrow metastasis, histological classification, histological type, and Children’s Oncology Group (COG) classification.

### 6.4. Breast Cancer

Multiple studies were conducted to study the effects of breast cancer-derived CAF exosomes on invasion and migration of breast cancer cells. In breast cancer tissues, Liu et al. found high levels of miR-3613-3p, an upregulated microRNA in CAFs exosomes shown to promote invasiveness by modulating SOCS2 expression [106]. Similarly, Yang et al. and Xiang et al. found that normal fibroblasts were transformed into CAFs by a microRNA, miR-146a, which in turn enhanced activation of the Wnt signal transduction pathway, promoting invasion and metastasis of breast cancer cells [38,107]. Enhanced invasion of breast cancer cells was also shown to correlate with increased levels of N-cadherin and vimentin in CAFs [38,107]. Studies by Wu et al. and Demircioglu et al. demonstrated that CAFs play a direct role in migration of breast cancer cells in vivo by modulating FAK signaling, as FAK depletion in CAFs increased chemokine production to activate protein kinase A, leading to enhanced malignant glycolysis [14,108].

### 6.5. Prostate Cancer

Shen et al. used IHC and found that Yes-associated protein 1 (YAP1) can convert normal fibroblasts into CAFs in the prostate cancer TME, which then exert several protumoral effects. YAP1 was upregulated in prostate cancer tissue, and FAP and α-SMA expression were significantly elevated in stromal and epithelial cells with high YAP1 expression levels, demonstrating the association between YAP1 and CAF development. Another study found that *p53S* induced overexpression of CAF-specific factors, such as α-SMA and vimentin, through the Stat3 pathway, resulting in accelerated prostate cancer cell development, migration, and invasion [33].

### 6.6. Renal Cell Carcinoma

FAP, a common CAF marker, was found to play a significant role in the propagation of CCRCC as it formed protein complexes with the urokinase plasminogen activator receptor (uPAR), promoting more aggressive tumor invasion. FAP expression was also correlated with synchronous lymph node metastases when examining a large array of patient samples [45,46]. With a focus on ECM POSTN, Bakhtyar et al. used IHC in xenograft models to illustrate that POSTN was found to coexist with α-SMA-expressing CAFs in the stroma of both local and metastasized CCRCC, indicating that CCRCC-associated POSTN was derived from stroma rather than tumors [109]. Likewise studying POSTN’s effects on RCC, Chuanyu et al. found that CAF-derived POSTN is a critical driver in RCC cell growth and migration through interaction with integrins αvβ3 and αvβ5, which activate the focal adhesion kinase/c-Jun N-terminal kinase pathway and enhance CCRCC cell attachment to increase metastatic capabilities.

### 6.7. Head and Neck Cancer

CAFs have been shown to be implicated in several different variants of head and neck cancers, most commonly NPC and head and neck squamous cell carcinomas (HNSCCs). Chen et al. identified α-SMA-positive CAFs in NPC, nasopharyngitis, and metastatic NPC tissues, and were able to determine a strong association between CAFs and the more malignant (more aggressive/invasive) phenotypes of NPC and metastatic NPC [110]. Further illustrating the role of CAFs in NPC, Zhu et al. showed that CAFs identified using IHC for α-SMA promoted upregulation of COX-2 expression in NPC patients with poor survival and distant metastases [10]. Importantly, COX-2 was highly expressed in CAFs even at the distant metastasis site, and inhibition of COX-2 and its downstream product prostaglandin E2 reversed the invasive and metastatic capacity in vitro, as did knockdown of COX-2 expression in vivo. The proposed mechanism is that COX-2 elevated TNF-α expression in CAFs to promote NPC cell migration, and the identification of this COX-2-mediated axis may provide opportunities for targeting CAFs in advanced NPC. In the context of HNSCC, Ramos-Vega et al. were able to demonstrate a significant association between α-SMA-positive CAF cells and laryngeal carcinomas, advanced clinical stages of HNSCC cancers, and lower tumor differentiation; there was, however, no observable association with PDPN staining and clinicopathological variables or presence of CAFs [7].

### 6.8. Bladder Cancer

According to Zhuang et al., mechanistically, TGF-β1 secreted by CAFs propagated cell invasion partially through increased expression of the ZEB2NAT/lncRNA-ZEB2 transcription factor axis in bladder cancer cells. CAFs in urinary bladder cancer were found to secrete numerous cytokines and inflammatory mediators, promoting invasion and metastasis [44]. Moreover, Zhou et al. demonstrated that higher expression of CAF-derived MFAP5 promotes malignant bladder cancer behavior through enhancement of the NOTCH2/HEY1 signaling pathway, resulting in increased invasion and propagation both in vivo and in vitro [111].

## 7. Effects of CAFs on Immune Cells in the Tumor Microenvironment

Modulation of the immune response by the tumor microenvironment is an aspect of tumor malignancy that has recently become more prevalent as the subject of several research studies. The role of stromal cells, and particularly CAFs in immunomodulation of cancer tissue, serves two primary purposes: CAFs facilitate tumorigenesis by inducing a chronic inflammatory state for cancer cells, and then it is also critical that in order for the tumor to survive, any immune response directed specifically towards the tumor must be mitigated [69,112]. With respect to the first component, the release of pro-inflammatory cytokines by CAFs can assist with the recruitment of macrophages, neutrophils, and lymphocytes to the tumor stroma. Within the TME, these cells can then differentiate into tumor-associated macrophages (TAMs) and tumor-associated neutrophils (TANs) and release several important endothelial factors and growth factors such as VEGF, HGF, MMPs, and various interleukins. Studies suggest that CAFs drive EMT and interact with M2 macrophages to promote the occurrence and progression of malignant tumors, as M2 macrophages are TAMs that engage in immunosuppression activities [113,114]. CAF contributions to immune invasion of tumor cells also offer an opportunity for a novel target in immunotherapy. 

### 7.1. Skin Cancer

Ersek et al. were able to determine that melanoma-associated fibroblasts (MAFs), or CAFs in melanoma cancer tissues identified by their FAP expression, impaired cytotoxic T lymphocyte function [42]. Their findings demonstrated that in the presence of MAF-conditioned media, cytotoxic T lymphocytes displayed dysregulated ERK1/2 and NF-κB signaling, impeded CD69 and granzyme B production, impaired killing activity, and upregulated expression of negative immune checkpoint receptors T cell immunoreceptor with Ig and ITIM domains (TIGIT) and B- and T- lymphocyte attenuator (BTLA). Compared to normal fibroblasts, MAFs displayed increased amounts of V-domain Ig suppressor of T cell activation (VISTA) and Herpesvirus entry mediator (HVEM), which are known ligands of BTLA, in addition to higher levels of SDF-1/CXCL12 release, which contributed to their interference of intracellular cytotoxic T lymphocyte signaling leading to anergy and dysregulation.

### 7.2. Head and Neck Cancer

In a study exploring the relationship between CAFs and TAMs in NPC, Yu et al. demonstrated that CAFs identified by IHC for positive α-SMA staining showed a strong positive correlation between density of CAFs and CD163-positive TAMs in pretreatment NPC tissues [115].

### 7.3. Lung Cancer

A study by Xiang et al. used mass cytometry to show that in patients with LSCC, there was a positive correlation between the CAF marker FAP and tumor-infiltrating immune cell (TIIC) markers, such as CD14, establishing a relationship between the presence of CAFs and their role in modulating the immune-evasive TME [4].

### 7.4. GI Cancer

In a study investigating the role of CAFs in immune suppression for PDAC, Fearon et al. demonstrated that the removal of FAP-positive CAF cells from the tumor in mice models enabled the immune control of tumor growth with anti-PDL-1 immunotherapy [116]. Further substantiating the role of CAFs in immune suppression, this study also showed that inhibition of SDF-1, of which CAFs are the only tumoral source, augmented the efficacy of immunotherapy and greatly diminished cancer cells. These findings provide a great scaffold for CAF-specific therapeutics, as FAP-positive cells are found in the vast majority of adenocarcinomas.

CAFs have also been found to promote an immunosuppressive TME in the context of OSCC, as described by Takahashi et al. [60]. CAFs were observed to affect the functional polarization of TAMs in vitro, with higher expression of CD68, CD14, CD163, CD200R, HLA-G, CD80, and CD86 in cancer cells co-cultured with CAF-conditioned media, as well as higher gene expression levels of ARG1, IL-10, and TGF-β1. They further demonstrated that cells in CAF-conditioned media exhibited suppression of T cell proliferation, and that this was reversible with the neutralization of TGF-β1 and IL-10. In addition to OSCC, CAFs have also been shown to affect the TME through immune modulation in esophageal cancers. In a recent study, Kato et al. demonstrated that CD8+ tumor-infiltrating lymphocyte levels and α-SMA-positive CAFs were negatively correlated, while FoxP3+ tumor-infiltrating lymphocyte levels and CAFs were positively correlated using both in vitro and in vivo models [117]. The study determined that CAF suppression of CD8+ and promotion of FoxP3+ tumor-infiltrating lymphocytes was mediated by IL-6, suggesting IL-6 blockade via CAF targeting as a possibility to enhance the efficacy of immunotherapy in esophageal cancers.

## 8. CAF Regulation of Cancer Cell Metabolism

Over the past few decades, metabolism in the context of cancer has gained considerable recognition due primarily to the pioneering efforts of Warburg et al., who first described the phenomenon of cancer cells consuming high levels of glucose in a process known as the Warburg effect [118]. Since then, studies have continued to increase our knowledge about cancer cell metabolic adaptations and modifications that enhance their ability to synthesize essential lipids, amino acids, and nucleotides to assist with malignant proliferation. Most recently, studies have shown that metabolic heterogeneity exists between tumors of the same subtype, as cancer cells have been shown to utilize sources other than glucose to metabolize carbon [119]. Concurrently, our increasing knowledge of cancer metabolomics has also led to the discovery of crosstalk between CAFs and cancer cells that contributes to the different metabolism programs across cancers.

One such example of how CAF cells may impact cancer cell metabolism was illustrated in a study by Pavlides et al., where the authors observed an upregulation of both myofibroblast markers and glycolytic enzymes (LDHA and PKM2) on proteomic and transcriptomic analysis on Cav-1-deficient stromal cells. This differential expression was found to be associated with tumor recurrence and poor prognosis in breast cancer patients [120]. On the basis of these observations, the authors postulated a synergistic effect where cancer cells stimulate aerobic glycolysis in neighboring CAFs, which then undergo myofibroblast differentiation and secrete high level of pyruvate and lactate to be used by the cancer cells as an energy source. Similarly, CAF cells isolated from breast cancer, colon carcinomas, melanoma, and PDAC were also shown to exhibit a Warburg effect characterized by increased glucose uptake and lactate production, with a corresponding decrease in oxygen consumption [121,122,123,124].

Aside from demonstrating an affinity for the Warburg effect, CAF cells have also been implicated with other metabolic processes including the use of alternative sources of carbon to provide energy for proliferation and growth. Comparisons between quiescent and proliferative fibroblasts demonstrated that only proliferating fibroblasts that are phenotypically representative of CAFs are able to use glutamine to replenish tricarboxylic acid (TCA) cycle intermediates [125]. CAFs isolated from PDAC were similarly shown to utilize glutamine as an alternate energy source, and were even further shown to be sensitive to glutamine deprivation, while remaining resistant to glucose starvation [124]. In ovarian carcinomas, two different SMA-positive CAF sub-populations (FAP-high, SMA-positive vs. FAP-neg-low, SMA-positive) were shown for the first time to harbor heterogeneous metabolic programming amongst CAF cells in the same tumor type [126]. In their study, one population of CAF cells exhibited increased expression of genes related to electron transport chain (ETC) complexes, suggesting an increased reliance on oxidative phosphorylation as compared to the other sub-population. This finding of heterogeneous metabolic programming was similarly found in head and neck cancer by RNA sequencing on isolated fibroblast cells [127].

The metabolic crosstalk between CAFs and tumor cells can result in many different synergistic effects including CAF support of cancer cell growth, proliferation, and migration/invasion by different metabolic mechanisms involving ECM stiffening, autophagy, and lipid secretion. Studies using PDX mouse models have been conducted to further investigate the underlying mechanisms of CAF/tumor cell crosstalk. As mentioned earlier, ovarian cancer cells act on surrounding fibroblasts to satisfy their demand for glutamine carbons to replenish TCA cycle substrates and support cancer growth by increasing purine and pyrimidine biosynthesis pathways; reciprocally, cancer cells provide lactate and glutamate to CAFs, enhancing the TCA cycle and allowing for increased glutamine production by CAFs [128]. ECM stiffening was also shown to exert metabolic reprogramming through a YAP/TAZ-dependent pathway to promote glycolysis and mitochondrial respiration in CAFs, whereas only glycolysis rates were shown to increase in cancer cells. This metabolic crosstalk promotes nucleotide biosynthesis and thereby enhances cancer cell proliferation [119]. Despite these important findings, many questions remain about how metabolomics can play a role in the development of novel therapeutics to suppress malignant evolution and proliferation of cancers cells, and will require extensive studies in the future.

## 9. CAF Effects on Therapeutic Resistance

**Figure 2:**
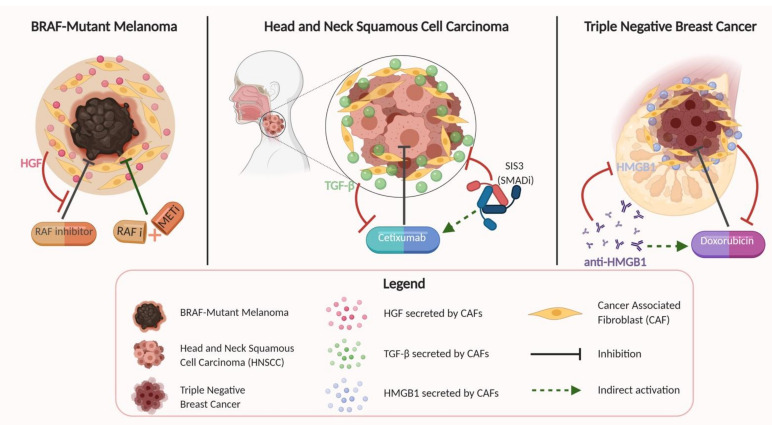
Here, we illustrate examples of mechanisms by which CAFs confer therapeutic resistance in three different cancer types. Therapeutic resistance can be promoted by secretion of hormones including hepatocyte growth factor (HGF) or transforming growth factor beta (TGF-β), or production of proteins such as high mobility group box 1 (HMGB1). Identification of these mechanisms and the responsible molecules may offer novel targets for combinatorial treatment regimens to combat or mitigate the development of therapeutic resistance.

One of the most intriguing aspects of CAFs and why they provide a unique opportunity for novel therapies is their contribution to therapeutic resistance in several different cancers (Figure 2).

Straussman et al. investigated several different cancer cell lines and stromal cell types, showing that specifically in BRAF-mutant melanoma cells, stroma-mediated resistance to RAF inhibition was conferred by stromal secretion of HGF, a commonly described product of CAF cells [129]. This resistance was reversed upon dual inhibition of RAF and MET, suggesting the possibility of dual treatment options for certain BRAF-mutant melanomas refractory to single therapy. Several other studies in the literature have explored the relationship between stromal cells such as CAFs and their ability to impart drug resistance in various cancers, including resistance to cetuximab in HNSCC [11]; resistance to 5-fluorouracil, epirubicin, and cyclophosphamide (FEC) therapy in estrogen receptor (ER)-negative breast cancer [13]; and doxorubicin resistance in triple-negative breast cancer [130]. The study conducted by Johansson et al. for HNSCC used a co-culture model similar to other reports in the literature to show that HNSCC and CAF co-cultured lines were resistant to cetuximab treatment, which is an EGFR antagonist. This effect persisted even when CAF-conditioned media was used for culturing HNSCC cells in a concentration-dependent manner, showing that CAFs were conferring this therapeutic resistance through soluble factors. Yegodayev et al. demonstrated a similar relationship between CAFs and mitigated efficacy of cetuximab in HNSCC, but also went further to show that TGF-β signaling was implicated in this association, and that blocking the TGF-β pathway using the SMAD3 inhibitor SIS3 enhanced cetuximab efficacy in PDX models. Guo et al. sought to explore the function of CAF-derived exosome microRNA-98-5p (miR-98-5p) in cisplatin resistance in ovarian cancer. Using immunofluorescence, they observed enhanced expression of specific CAF marker proteins (α-SMA, FAP, and FSP1) in the stroma and found an association between CAF-derived miR-98-5p and suppression of cyclin-dependent kinase inhibitor 1A (CDKN1A) as a downstream target. Knocking out CDKN1A promoted cisplatin resistance in ovarian cancer cells, increasing ovarian cancer cell proliferation and decreasing apoptosis in previously cisplatin-sensitive cells. This study helped illustrate a potential mechanism by which CAFs promote therapeutic resistance via miR-98-5p-mediated CDKN1A inhibition in ovarian cancer [48].

With respect to breast cancer, Farmer et al. demonstrated using gene expression data sets that increased stromal gene expression predicted resistance to FEC therapy in ER-negative breast cancer tumor biopsies, with validation in independent, external datasets. Amornsupak et al. demonstrated CAF-conferred resistance to doxorubicin in triple-negative breast cancer cells using similarly constructed co-culture and conditioned media experiments [130]. Their study showed that CAF cells were able to induce resistance to doxorubicin through high mobility group box 1 (HMGB1) production, as pre-treatment of breast cancer cells with CAF-conditioned media induced a degree of resistance to doxorubicin that corresponded to levels of secreted HMGB1, compared to negligible resistance in non-tumor-associated fibroblast-conditioned media. Additionally, this result of induced resistance was recapitulated with exogenous recombinant HMGB1 addition, and resistance could be significantly reduced with application of anti-HMGB1 antibodies.

Pancreatic cancer has also been the subject of several studies investigating the role of CAFs in therapeutic resistance. With pancreatic cancer, however, the relationship between CAFs and their secretory products with cancer cells is more complex [131]. Ozdemir et al. conducted a novel study exploring the effects of CAF cell depletion in the context of PDAC, finding that depletion of α-SMA-positive cells at either non-invasive precursor or the PDAC stage led to invasive, undifferentiated tumors with enhanced hypoxia and EMT, with diminished overall survival [132]. While CAF-depleted tumors did not respond to gemcitabine, anti-CTLA4 immunotherapy reversed the disease acceleration and prolonged animal survival. This study serves to highlight the need to carefully interrogate the role of CAFs in different cancer contexts in order to better understand how potential therapeutics can be beneficially applied. In the context of gastric cancers, Zhang et al. showed that CAFs secrete exosomal miR-522, which inhibits ferroptosis in cancer cells by targeting ALOX15, blocking accumulation of toxic lipid peroxides and thereby preventing non-apoptotic cell death by ferroptosis [23].

## 10. CAF Subtypes

Given the marked heterogeneity present in CAF markers, several studies have begun to postulate that CAFs identified by a single marker, or even CAFs identified by different markers, may actually compose a range of distinct CAF subtypes that have functionally different roles in cancer progression [1]. Accordingly, it maybe become necessary to subdivide CAFs on the basis of a combination of several marker proteins in order to better characterize their unique biology and corresponding therapeutic relevance to cancer. For example, multicolor flow cytometry has identified four CAF subtypes in breast cancer, each with distinct interactions with tumor T cells [133], while analysis of breast cancer CAFs along the whole transcriptome has suggested three CAF subtypes [134]. The three subsets of CAFs in breast cancer identified using single-cell RNA sequencing included dCAFs (discriminated by expression of genes involved in development and morphogenesis), mCAFs (discriminated by expression of genes involved in ECM and EMT), and vCAFs (discriminated by expression of genes involved in vascular development and angiogenesis). With respect to colorectal cancer, Li et al. demonstrated once again using single-cell RNA sequencing that CAFs associated with colorectal cancer samples exhibited two major subtypes: CAF-As and CAF-Bs. CAF-As were characterized by their high expression of MMP2, decorin, COL1A2, and FAP, while CAF-Bs were characterized by more typical myofibroblast markers such as α-SMA, transgelin, and PDGF-α. The diversity amongst CAFs suggests that some functionally heterogeneous subsets may even promote or restrain cancer growth [73].

## 11. Utility of CAFs as Prognostic Markers

In addition to the diverse range of CAF phenotypes, the presence of different CAF markers as well as CAF density in histopathological tissues have been implicated as prognostic markers in myriad cancers. With respect to GU cancers, Colvin et al. found that increased differential expression of nine large non-coding RNAs (lncRNAs) and decreased expression of one lncRNA identified in ovarian CAFs were associated with decreased overall survival in ovarian cancer patients and can potentially be utilized as targets to regulate CAF function [25]. Similarly, Mezheyeuski et al. demonstrated that increased expression of FAP and PDGFR-β CAF markers was significantly associated with decreased survival in analysis of urothelial bladder cancer patient samples, and that FAP expression was a significant independent prognostic marker when analyzing different patient clusters [43]. Moving past GU cancers, several studies showed similar utility of CAF markers in breast, skin, and head and neck cancers as well. Using IHC, researchers found that stromal expression of CAF-related proteins such as PDPN, FAP, PDGFR-α, and PDGFR-β was significantly elevated in the stroma of metastatic breast cancer tissue (*p* < 0.005), being found to be correlated with reduced survival from breast cancer using several different breast cancer patient samples [86,135]. In NMSC patient samples, the overexpression of mDia2 in stromal cells as a result of high levels of activin A correlated highly with increasing tumor malignancy and decreased survival in NMSC patient samples [93]. This study demonstrated the novel pathway of cancer cell activin A promoting CAF cell differentiation through activation of the Smad2–mDia2–p53 signaling axis and how expression of these markers significantly associated with patient survival and NMSC malignancy. In head and neck cancer, Yu et al. found that α-SMA and CD163 expression were both independent predictors of survival in patients with NPC through Cox multivariable analysis and Kaplan–Meier analysis, demonstrating how different components of the TME can influence malignancy and overall patient survival [115]. Additionally, Chen et al. showed that density of CAFs (identified by α-SMA expression) in NPC and metastatic NPC tissues was highly correlated with tumor T stage and relapse, with a significant difference in overall survival as well between high-density (worse survival) and low-density CAF tissues on Kaplan–Meier analysis. Further, multivariate Cox analysis demonstrated that CAF density in tumor tissue could be used as an independent prognostic factor for survival in NPC patients [110].

Several studies have also been conducted in the context of GI cancers in order to better understand how the presence of CAFs in the TME may offer prognostic utility when assessing patient survival. Sha et al. showed that in ICC tumor CAF expression of α-SMA was significantly correlated with tumor size, tumor numbers, lymph node metastasis, and advanced histological grade in ICC patients, as well as statistically significant independent predictors of poor overall and recurrence-free survival [54,57]. In gastric adenocarcinoma, Cong et al. demonstrated that CAFs identified by IHC staining for α-SMA and TANs assessed by IHC staining for CD66b could be used as independent factors for patient outcomes and to identify patients who might benefit from postoperative chemotherapy [136]. Their study illustrated a strong correlation between the presence of TANs and CAFs with poorer disease-free survival, but notably also showed a phenotypic advantage in CAF/TAN-poor cancers treated with postoperative chemotherapeutics. When exploring oral cancers, Wang et al. demonstrated that the CAF marker FAP was highly expressed in carcinoma cells of OSCC, and that high expression levels of FAP correlated closely with poor overall survival [61]. Similarly, in a study by Takahashi et al., CAFs were found to be independent prognostic factors in OSCC, and correlated with lymphatic invasion, vascular invasion, lymph node involvement, and TNM staging [60]. In summation, these studies show the potential role CAFs play in prognosticating patient survival, given their integral role in promoting tumor malignancy through a variety of mechanisms.

## 12. Potential Therapeutic Targets for CAFs

### 12.1. Lung Cancer

In recent studies, FAP has been considered a target for therapeutic intervention in lung cancer. According to Santos et al.’s study on xenograft models of lung cancer, administering a new anti-FAP monoclonal antibody, FAP5-DM1, can either obstruct tumor growth for an extended time period or completely inhibit the tumor, causing tumor regression. However, due to FAP’s non-specific expression on CAFs, there may be potential therapeutic side effects from immunotherapy targeting FAP [16]. According to Li et al., FUT8 has also been found to be a potential therapeutic target of NSCLC [15]. Furthermore, inhibiting VCAM-1, a common product of CAFs, reduced the proliferation and invasion of lung cancer cells, implying that VCAM-1 can be further explored as a potential therapeutic target [84]. Due to the difficulty in translating an anti-matrix therapy into clinical practice, there is an implication that targeting the cell matrix alone may not be enough. Targeting tumor parenchymal cells and stromal cells concurrently may be more successful to diminish protumoral effects in lung cancer (Table 2) [105].

### 12.2. GI Cancer

Studies have well-characterized the role of CAFs in ICC tumors, and Yamanaka et al. demonstrated that administration of nintedanib (tyrosine-kinase inhibitor targeting VEGFR, PDGFR, and FGFR) could suppress the protumoral effects of CAFs derived from ICC tissues both in vitro and in vivo [141]. Nintedanib is an anti-fibrotic drug that suppressed CAFs expressing α-SMA and greatly reduced levels of ICC-promoting cytokines IL-6 and IL-8, secreted by CAFs. In vivo studies with nintedanib demonstrated reduced xenograft growth as well as reduced number of activated CAFs expressing α-SMA, and that combination therapy with nintedanib and gemcitabine against CAFs and ICC cells had the strongest inhibition of tumor growth. In the context of colorectal cancers, CAFs have long been implicated in promoting tumor growth both directly as well as indirectly. In a study by Fourniols et al., the efficacy of paclitaxel (PTC) and ariflavine (ACF) were tested as potential inhibitors of CAF development [142]. Using novel lipid nanocapsule formulations (LNC), they were able to demonstrate CAF inhibition with use of LNC-ACF, and whole tumor inhibition by LNC-PTX, offering a potentially novel strategy for reducing CAF populations in the colorectal TME, thus mitigating tumor growth.

### 12.3. Breast Cancer

In addressing therapeutic implications for breast cancer, several studies have found CAF-targeted approaches that are now emerging as potential strategies to treat various types of breast cancer [35,143]. Studies have elicited that FAP-expressing CAFs can demonstrate tumor-forming functions in some cancers such as gastric cancer, but also asporin-expressing CAFs were found to inhibit cancer development in breast cancer, offering two different opportunities for therapeutic intervention [86]. Additionally, it was demonstrated that silencing β1-integrin, a target gene of G protein-coupled ER in tamoxifen-resistant cells, potentially reduces cell migration and EMT of cancer cells mediated by CAFs [86,144]. To therapeutically target CAFs, Geng et al. treated 4T1 breast cancer model mice with an FAPα-targeted DNA vaccine, which led to tumor regression, reduced CAF expression, and diminished secretion of SDF-1 and CCL2 [86,138]. Through ex vivo and in vivo assays, Shu et al. found increased expression of α-SMA, PDGFR-α, FAP, and TGF-β in C3a gene-treated CAFs, highlighting the *C3aR* gene as a potential therapeutic target to control tumor metastasis in breast cancer [145]. Moreover, according to Liu et al., when miR-3613-3p was downregulated in the CAFs exosomes, SOCS2 expression (a suppressor of cytokine signaling 2) was targeted and breast cancer cell metastasis was diminished, suggesting that miR-3613-3p functions as a potential therapeutic target [106]. Although current anti-breast cancer therapy targets have not been efficient due to the complexity of the TME, further investigation of the outlined potential target pathways may lead to tangible therapeutic strategies.

### 12.4. Skin Cancer

In a unique approach to investigating the role of CAFs in cutaneous squamous cell carcinoma, Nie et al. showed that CAFs are integral to the promotion of an acute inflammatory reaction in aminolaevulinic–photodocynamic therapy (ALA-PDT) [41]. Through use of anti-IL1β monoclonal antibodies, Nie et al. demonstrated that PDT stimulates a local inflammatory response in cancer cells through activation of NLRP3 inflammosome with synergistic IL1β production from CAFs. This novel insight shows how CAFs may also be used to augment our ability to neutralize cancer cells either by harnessing their ability to induce inflammatory responses, or by mitigating their protumoral effects along specific axes.

### 12.5. Ovarian Cancer

According to Sun et al., although FGF-1’s specific mechanistic function and role in tumorigenesis is still unclear, FGF-1 secreted by CAFs can be a potential target for the treatment of ovarian cancer given its role in enhancing FGF-1/FGFR4 signaling to promote tumor cell proliferation, migration, and invasion [47]. Using a different approach, autophagy, which is an essential part of tumor metabolism that protects ovarian CAFs against oxidative stress, was also found to be a potential therapeutic option as targeting maternally expressed 3 (*MEG3)* and metastasis associated lung adenocarcionoma transcript 1 (*MALAT1)* lncRNAs that modulate autophagy can potentially diminish CAF and ovarian cancer progression [25].

### 12.6. Endometrial Cancer

Teng et al. found that CAFs enhance endometrial cancer cell proliferation, migration, and invasion in both in vivo and in vitro studies through SDF-1 secretion and subsequent enhancement of the PI3K/Akt and MAPk/Erk signaling pathways. Addition of CXCR4 antagonists to co-cultured studies inhibited these protumoral effects by suppressing the SDF-1α/CXCR4 axis, offering a potential target for therapy in endometrial cancer patients [26].

### 12.7. Prostate Cancer

According to studies by Ortiz-Otero et al., CAFs provide resistance to prostate tumor cells and can thus be a target for novel prostate cancer therapeutics [32]. Silencing YAP1 being expressed by CAFs and tumor stromal cells effectively inhibited tumor growth in their study, implying its potency as a therapeutic target [34]. Neuwirt et al. also found a promising mechanistic pathway for inhibiting therapy resistance in prostate cancer relating to CAF-mediated upregulation of cholesterol and steroid biosynthesis, which cause tumor growth and progression while resisting androgen receptor (AR)-targeted therapies. However, if resistance pathways are repressed with simvastatin and an AKR1C3 inhibitor, this can likely overcome resistances to AR targeted therapies in prostate cancer [139]. 

### 12.8. Bladder Cancer

Studies by Zhou et al. show that when CAF secretion of MFAP5 is downregulated, the invasion and migration of bladder cancer cells is inhibited by suppressing the NOTCH2/HEY1 signaling pathway. Targeting MFAP5 can prove to be a new diagnostic and therapeutic method for bladder cancer treatment [111].

## 13. Conclusions

In summation, CAFs represent a unique and clinically relevant component of the TME that are integral to better understanding the complex interplay between cells in the TME, the ECM, and cancer cells themselves in different cancer contexts. The functional and phenotypical heterogeneity inherent to CAFs presents a challenge as well as an opportunity to incorporate more specific and personalized therapies as they pertain to individual patients and cancers. In earlier studies, several canonical markers for CAFs were identified to help distinguish them from normal fibroblasts, but the identification of a single defining marker never came to fruition. Instead, we now understand that CAFs activated in the tumor stroma can adopt a wide variety of markers, and that different combinations of expressed markers may actually yield further insight into functional CAF subtypes. Despite these challenges, it is undeniable that CAFs are implicated in several different aspects of tumor progression and are an integral part of stromal support provided to cancers. From maintenance of stemness, to promotion of angiogenesis and metastatic invasion, CAFs have been firmly established as potential targets for the continued advancement of cancer therapies. As such, additional studies, particularly utilizing newly developed bioinformatics techniques in combination with basic science research, will be imperative to further our understanding of this critical component of the TME.

## Figures and Tables

**Table 1:** Summary table of different cancer types, and the markers that have been used to identify cancer-associated fibroblasts (CAFs) within the context of their respective tumor microenvironments.

Cancer Type	Markers	Relevant Studies
Breast	α-SMA, FAP, PDGFRα, PDGFRβ, CD29, NG2, FSP1, vimentin, PDPN	Jung et al. (2015) [35], Aboussekhra et al. (2011) [36], Pelon et al. (2020) [37], Yang et al. (2020) [38]
Lung	α-SMA, FAP, vimentin, PDGFRβ, CD90, PDPN	Fan et al. (2020) [39], Xiang et al. (2020) [4], Neri et al. (2015) [40]
Skin	α-SMA, FAP, vimentin, PDGFRα	Nie et al. (2019) [41], Ersek et al. (2020) [42], Erez et al. (2010) [20]
Genitourinary: Bladder	α-SMA, FAP, CD90, vimentin, PDGFRα, PDGFRβ, MFAP5, FSP1	Mezheyeuski et al. (2020) [43], Zhuang et al. (2015) [44]
Genitourinary: Prostate	α-SMA, vimentin, FAP, FSP1, PDGFR-α, PDGFRβ	Ortiz-Otero et al. (2020) [32], Liu et al. (2020) [33], Shen et al. (2020) [34]
Genitourinary: Renal	α-SMA, FAP, POSTN	Errarte et al. (2020) [45], Zagzag et al. (2005) [46]
Genitourinary: Ovarian	α-SMA, FAP, FSP1, FGF-1	Sun et al. (2017) [47], Guo et al. (2019) [48]
Genitourinary: Endometrial	α-SMA, FSP1, FAP, vimentin	Teng et al. (2016) [26]
Gastrointestinal: Colorectal	FAP, α-SMA, vimentin, FSP1, PDGFR-α	Herrera et al. (2013) [49], Bai et al. (2015) [24]
Gastrointestinal: Esophageal	vimentin, a-SMA	Zhao et al. (2020) [50]
Gastrointestinal: Gastric	FAP, α-SMA, FSP-1, vimentin, PDGRFα, PDGFRβ	Zhang et al. (2020) [23], Shi et al. (2020) [51], Ham et al. (2019) [52]
Gastrointestinal: Pancreatic	α-SMA, vimentin, FAP, PDGFRβ, FSP1, PDGFR-α	Zhang et al. (2020) [23], Wei et al. (2018) [53]
Gastrointestinal: Liver and Biliary System	α-SMA, FAP, FSP1, PDGFR-β, periostin	Chuaysri et al. (2009) [54], Zou et al. (2018) [55], Lau et al. (2016) [56], Sha et al. (2018) [57], Affo et al. (2017) [58]
Gastrointestinal: Oral	FAP, α-SMA, vimentin	Bello et al. (2011) [59], Takahashi et al. (2017) [60], Wang et al. (2014) [61]
Head and Neck	α-SMA, PDPN, FAP, PDGFR-α, PDGFR-β, FSP1, NG2	Ramos-Vega et al. (2020) [7], Zhu et al. (2020) [10], Wang et al. (2014) [62]
Endocrine/Neuroendocrine	A-SMA, FAP	Hashimoto et al. (2016) [63], Minna et al. (2020) [64]

α-SMA—alpha smooth muscle actin, FAP—fibroblast activation protein, PDGFRα—platelet derived growth factor receptor alpha, PDGFRβ—platelet derived growth factor receptor beta, NG2—neural/glial antigen 2, FSP1—fibroblast specific protein 1, PDPN—podoplanin, MFAP5—microfibrillar associated protein 5, POSTN—periostin, FGF-1—fibroblast growth factor 1.

**Table 2:** Summary table of CAF markers or their secretory products that have been tested either in clinical trials or pre-clinical studies as possible therapeutic targets.

Gene/Marker	Drug	Observed Effect
SDF-1/CXCR4 axis	AMD3100 (Plerixafor) “an anti-SDF-1 neutralizing antibody” (clinical trial) [26,137]	Reversed FAP-positive AF-mediated immunosuppression; decreased proliferation, migration, and invasion as well as in vivo tumorigenesis
TGF-β	Tranilast (Rizaben) (clinical trial) [137]	Inhibited TGF-β signaling in CAFs, facilitating T cell penetration into the tumor nest, and promoting anti-tumor immunity and tumor regression
IL-6	ROCKs and STAT3 “inhibitors of IL-6” (clinical trial) [137]	Improved the anti-tumor immune response and treated myeloproliferative diseases and autoimmune disorders
FAP	FAP5-DM “anti-FAP monoclonal antibody” (pre-clinical study) [16]; “FAPα-targeted DNA vaccine” (pre-clinical study) [86,138]; Sibrotuzumab “anti-FAP monoclonal antibody” (clinical trial) [69]	Obstructed tumor growth for an extended time period or completely inhibited the tumor causing tumor regression; diminished secretion of SDF-1 and CCL2
Tenascin-C	81C6 “anti-tenascin monoclonal antibody” (clinical trial) [69]	Targeted Tenascin-C, decreasing progression of colon cancer metastasis by mitigating response to TGF-beta signaling
Androgen receptor	Simvastatin and AKR1C3 inhibitor (pre-clinical study) [139]	Overcame resistance to androgen receptor-targeted therapies in prostate cancer; enhanced tumor regression with targeted treatment
Hedgehog signaling	Smoothened (SMO) inhibitor (clinical trial) [140]	Blocked Hedgehog signaling pathway activated in CAFs that fuels the therapy resistant phenotype in tumor cells
